# Complete Genome Sequence of *Psychrobacter* sp. Strain KH172YL61, Isolated from Deep-Sea Sediments in the Nankai Trough, Japan

**DOI:** 10.1128/MRA.00326-19

**Published:** 2019-04-18

**Authors:** Daniel Evans-Yamamoto, Nao Takeuchi, Takahiro Masuda, Yumi Murai, Yasuhide Onuma, Hideto Mori, Nanami Masuyama, Soh Ishiguro, Nozomu Yachie, Kazuharu Arakawa

**Affiliations:** a Institute for Advanced Biosciences, Keio University, Tsuruoka, Japan; b Systems Biology Program, Graduate School of Media and Governance, Keio University, Fujisawa, Japan; c Synthetic Biology Division, Research Center for Advanced Science and Technology, The University of Tokyo, Tokyo, Japan; d Faculty of Environment and Information Studies, Keio University, Fujisawa, Japan; e Department of Biological Sciences, School of Science, The University of Tokyo, Tokyo, Japan; f PRESTO, Japan Science and Technology Agency (JST), Tokyo, Japan; Georgia Institute of Technology

## Abstract

*Psychrobacter* sp. strain KH172YL61 is a Gram-negative bacterium isolated from deep-sea sediment in the Nankai Trough in Japan. Here, we report the complete genome sequence of this strain, which has a genome size of 3.19 Mb, with a G+C content of 44.0%.

## ANNOUNCEMENT

The genus *Psychrobacter* is a group of Gram-negative, psychrotolerant, aerobic, and nonmotile bacteria (1). They are able to grow below 5°C and have been isolated from various cold habitats. To date, strains of this genus have been isolated from low-temperature environments, including deep-sea sediments, Antarctic soil, and sea ice (2). Some *Psychrobacter* strains produce carbonic anhydrase enzymes, which suggests their potential for bioremediation through precipitation of heavy metals (3). Several *Psychrobacter* strains are reported to produce cold-adapted enzymes (4, 5). The diversity and high catalytic activity of strains as well as low energy consumption at low temperatures support great potential for further exploration of the genus.

*Psychrobacter* sp. strain KH172YL61 was isolated from deep-sea sediment collected 3,308 m below sea level in the Nankai Trough in Japan (33°27′005″N, 137°16′990″E). The taxonomy was assigned by Sanger sequencing of amplified 16S rRNA genes, for which BLAST search matched with 100% identity to many sequences from the *Psychrobacter* genus. A frozen sample was used to inoculate a modified seawater broth (360.75 mM NaCl, 7.5 mM KCl, 8.25 mM CaCl_2_·2H_2_O, 18 mM MgCl_2_·6H_2_O, 0.75 mM NaHCO_3_, 10.5 mM MgSO_4_·7H_2_O, 5.0% [wt/vol] Bacto peptone, 3.0% [wt/vol] yeast extract, and 1.5% Bacto agar) plate for the isolation of single colonies. A single colony was then used to inoculate an overnight culture in the modified artificial seawater broth (30°C with shaking at 160 rpm). The total genomic DNA was isolated using the Genomic-tip 20/G system (Qiagen). A genomic DNA library for sequencing was prepared using the rapid barcoding kit (SQK-RAB004) and sequenced on a GridION device with a FLO-MIN106 flow cell (Oxford Nanopore Technologies). Reads with at least 10,000 bp were used for the *de novo* assembly (86-fold coverage, 28,000 out of 111,000 reads) using Canu version 1.8.0 (6). Contigs obtained from the assembly were polished using Nanopolish version 0.10.1 (7). The resulting genome sequence was functionally annotated using DFAST version 1.0.2 (8). The assembled genome consists of one circular chromosome of 3,188,207 bp having 44.0% G+C content, including 4,129 coding sequences (CDSs), 50 tRNAs, and 15 rRNAs, respectively. Assessment of the genome completeness using gVolante version 1.2.0 (9) showed 85% BUSCO completeness, a quantitative assessment of assembly completeness based on the coverage of an evolutionarily conserved set of single-copy orthologs, indicating a certain amount of uncorrected indels. All software programs were used with the default settings.

According to the annotation results, the genome of *Psychrobacter* sp. KH172YL61 encodes genes involved in the production of cold shock proteins, namely *cspA* and *cspV* (10). Cold shock proteins are known to counteract cold stress conditions by serving as nucleic acid chaperons. The complete genome reported in this work may facilitate the understanding of mechanisms of cold stress response within psychrophiles.

### Data availability.

The chromosome sequence reported here was deposited in DDBJ/GenBank under accession number AP019516 and in the Sequence Read Archive (SRA) under accession number PRJNA521446.
